# Supplementary material for: Mutations in PCYT2 disrupt etherlipid biosynthesis and cause a complex hereditary spastic paraplegia
Source: Brain. 2019 Oct 22;142(11):3382–97. doi: 10.1093/brain/awz291 (PMC6821184; doi:10.1093/brain/awz291)
Supplement: awz291_Supplementary_Data [file awz291_supplementary_data.zip › awz291-Suppl_data/Supplementary_Data4.zip!/Supplementary information 4 - plasma lipidomics.zip!/Plasma lipidomics/Control vs Patient3b/Statistical plots and data/PS species/Volcano plot_alternative.pdf]

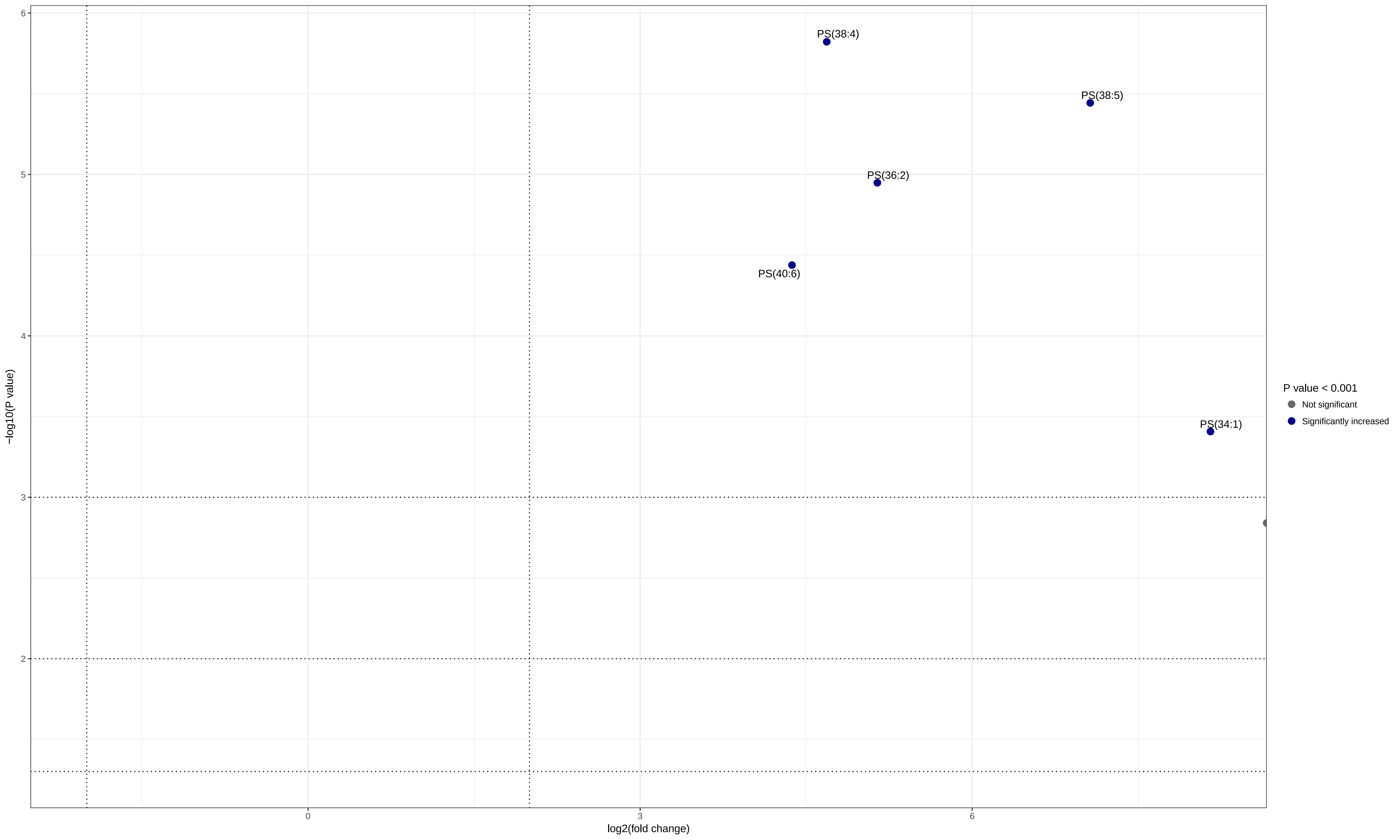
PS(38:4)
PS(38:5)
PS(36:2)
PS(40:6)
PS(34:1)
P value < 0.001
Not significant
Significantly increased
−log10(P value)
log2(fold change)
6
5
4
3
2
0
3
6
